# A feasibility study of a physical activity intervention for people with severe mental illness in medium secure psychiatric services in the United Kingdom

**DOI:** 10.3389/fpsyt.2025.1558602

**Published:** 2025-07-24

**Authors:** Gloria Roden-Lui, Guy Faulkner, Mike Lucock, Simon Gibbon, Catherine Hewitt, Elizabeth Hughes, Wajid Khan, Kiara Lewis, Bal Singh, Phil Walters, Judith Watson, Tammi Walker

**Affiliations:** ^1^ Department of Psychology, Durham University, Durham, United Kingdom; ^2^ Faculty of Education, University of British Columbia, Vancouver, BC, Canada; ^3^ Department of Nursing, University of Huddersfield, Huddersfield, United Kingdom; ^4^ Arnold Lodge, Nottinghamshire Healthcare NHS Foundation Trust, Nottingham, England, United Kingdom; ^5^ York Trials Unit, University of York, York, United Kingdom; ^6^ Department of Nursing and Community Health, Glasgow Caledonian University, Glasgow, United Kingdom; ^7^ South West Yorkshire Partnership NHS Foundation Trust, Wakefield, United Kingdom; ^8^ Centre for Life and Sport Sciences, Birmingham City University, Birmingham, United Kingdom; ^9^ Creative Minds Charity, South West Yorkshire Partnership NHS Foundation Trust, Wakefield, United Kingdom

**Keywords:** physical activity, severe mental illness, co-production, intervention design, COM-B model of behaviour, secure psychiatric services, feasibility and acceptability

## Abstract

**Background:**

In the UK, there are approximately 3500 individuals detained in medium secure service. Service users in such settings have complex and severe mental illness (SMI), often with co-morbid physical health problems, shorter life expectancy and low levels of physical activity (PA). However, there are few studies about PA interventions for medium secure service users in the United Kingdom. Therefore, the aim of the study was to co-produce, with medium secure service users and staff, the content and delivery of an intervention to increase PA.

**Methods:**

A feasibility and acceptability study were conducted to test the PA intervention using the Capability, Opportunity, Motivation Behaviour Change Model (COM-B model) as the underpinning theoretical model. Both female and male service users, with personality disorder and/or mental illness, were recruited from two medium secure services in the UK.

Outcome measures were collected at baseline, after the intervention and at follow up (3-months after intervention), and included PA levels, mental well-being, data on recruitment and retention and causes of drop out.

**Results:**

A total of 33 participants were recruited. Seven participants withdrew during the intervention period and 26 participants completed the PA intervention. During the follow up stage, three participants withdrew. Following the intervention participants increased PA and improved physical health and wellbeing. Overall, there was good retention for the PA intervention

**Conclusion:**

Retention rates and completeness of data at both study sites indicate that it is feasible and acceptable to co-produce, deliver and maintain commitment to a PA intervention in such settings for service users with SMI. A future pilot randomised controlled trial (RCT) will allow further understanding about the effectiveness of the PA intervention in medium secure psychiatric services.

**Clinical trial registration:**

https://doi.org/10.1186/ISRCTN15546527, ISRCTN Registry – ISRCTN15546527.

## Introduction

People with severe mental health illnesses (SMI), including schizophrenia, bipolar disorder and major depression, experience significant health disparities such as cardiometabolic diseases (e.g., obesity and hypertension) being twofold higher and premature mortality by 10—30 years (1–3). Regular physical activity (PA) can have a beneficial impact in both the general population and those with SMI and improve mental health (4, 5) and reduce the risk of cardiometabolic disease (6). However, people living with SMI are less likely to engage in moderate exercise and more likely to engage in sedentary behaviour than the general population (7, 8).

People with SMI, particularly those residing in medium secure psychiatric services, find it very difficult to maintain a healthy lifestyle (9). This type of service provides care and treatment to adults with severe mental health problems, who present a serious risk of harm to others and to themselves, and who are prevented from leaving hospital (Mental Health Act 1983, amended in 2007). Currently there are approximately 6000 people detained in mental health services in the UK, of which 3500 are at a medium secure level (10). Service users in such settings, compared to their non-secure counterparts, find it difficult to maintain a healthy lifestyle because the restricted environment limits access to exercise due to issues of risk and patient safety (9) and they are not free to leave (11). Additionally, mental health symptoms, medication side effects such as fatigue, and inconsistent attitudes and views towards exercise by staff are further barriers to exercise (12).

Limited PA is recognised as a major health problem for people with SMI in secure services (13). However, there is currently no national model for providing physical health interventions in routine care in such mental health services and physical health interventions are not routinely delivered. A review of physical activity in mental health settings (14), found only 3 studies within secure settings. Whilst these studies found improvements in symptoms the lack of studies and quality of the research prompted calls for further research in this area. Kinnafick et al. (2018) (15) interviewed health care assistants within secure settings and found conflicting views towards physical activity and Rogers et al. (2021) (12) similarly have reported a culture of inactivity within secure mental health settings because of both environmental and personal barriers.

The National Institute for Health Research (NIHR) commissioned a feasibility study (RfPB 201176) to co-produce, with medium secure service users, the content and delivery of an intervention to increase PA As there has been no previous trials of this nature in the United Kingdom (UK), the first step in the process for evaluation of a complex intervention in this setting was to assess feasibility and acceptability to establish the parameters for a future pilot randomised controlled trial (RCT). As the first such study we set more flexible feasibility goals in terms of a) identifying whether we could recruit participants, b) if participants would engage with the intervention, c) if there was some indication that the intervention was associated with a change in physical activity, and d) whether the intervention was described as acceptable by patients and staff.

## Materials and methods

### Study design

The IMPACT study was a quasi-experimental one arm, pre-test post-test feasibility study design. The research team assessed and collected participants’ PA levels and clinical outcome measures at baseline (prior to the intervention), post intervention (after the intervention) and at a follow up stage (three months post intervention).

### Setting

The study took place in two UK National Health Service (NHS) medium secure psychiatric services (Study Site A and Study Site B), which provide inpatient treatment and care to adult service users with severe mental health problems who present a serious risk of harm to others and/or to themselves. Service users in these settings are detained under the Mental Health Act 1983 (amended in 2007) and are prevented from leaving the hospital without authorisation from their Responsible Clinician.

Study Site A was based in a mixed rural and urban area in the north of England, UK and had 90 beds and 7 wards. Study Site B was based in a city in the Midlands of England, UK and had 102 beds and 6 wards. Both study sites serve a regional population and have corresponding catchment areas that cater to service users from the surrounding rural and urban areas.

### Sample size

The sample size calculations were based on estimating attrition, but no formal sample size calculation was undertaken as the main purpose of this study was to refine study procedures and the PA intervention for testing in a future pilot RCT study. The research team identified that a minimum of 15–20 participants in each study site would meet the objectives of testing the PA intervention (16, 17).

## Recruitment

### Participant eligibility

Inclusion Criteria.

- aged 18 and over;
- on a medium secure ward and stay in the hospital for the next 6 months;
- diagnosed with a severe mental illness;
- willing and able to provide written informed consent.

Exclusion Criteria.

- lacking capacity to consent (as guided by the Mental Health Act 1983 (amended in 2007);
- who are too acutely unwell and/or having a severe physical illness that precluded them from active participation;
- who pose a significant risk to self-and/or others;
- a non-English speaker (adapting the intervention is currently beyond the scope of this study);
- have a learning disability.

### Recruitment into the study

**Figure 1:**
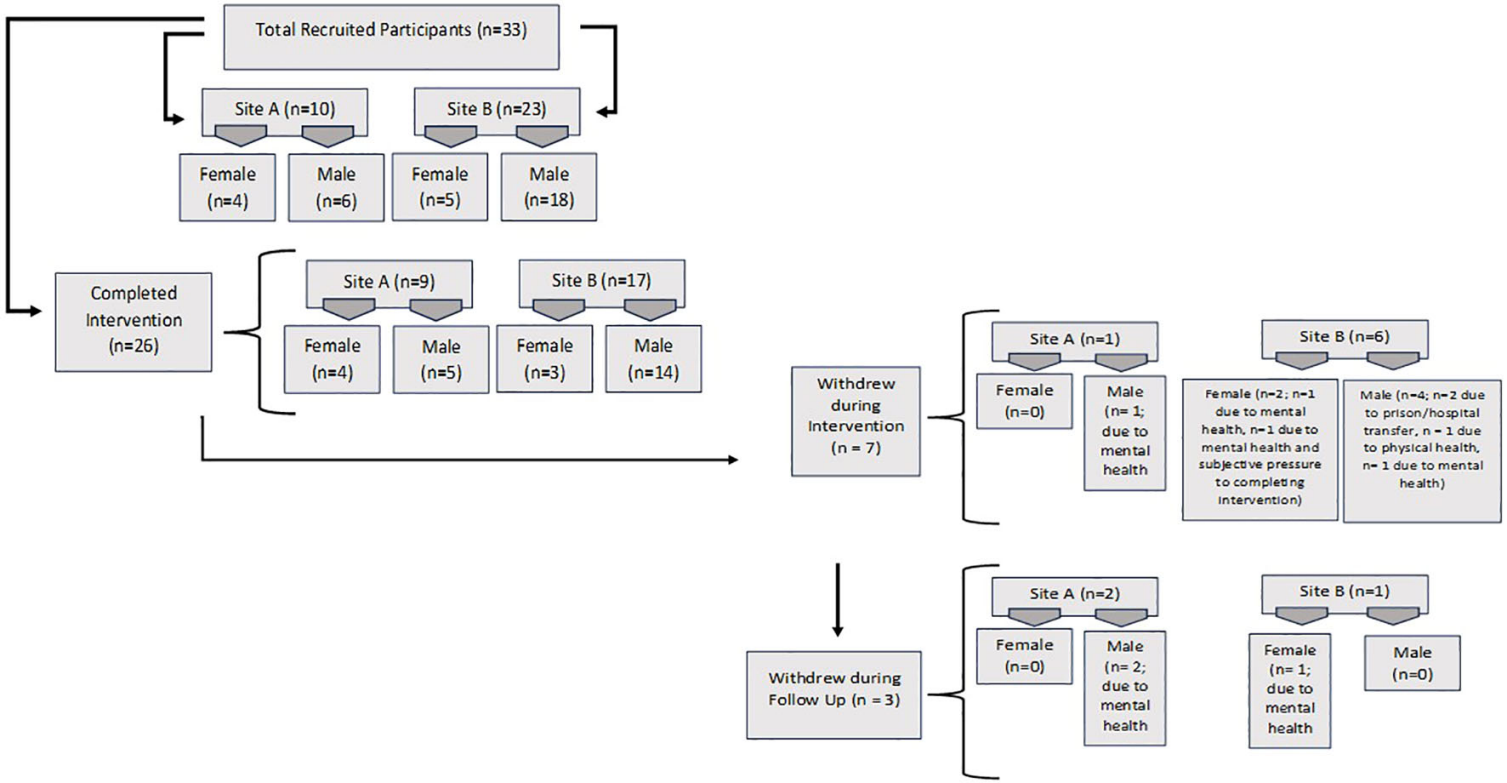
Recruitment and withdrawal rates here.

In total, the research team recruited 10 service users as participants at Study Site A and 23 service users as participants at Study Site B (n=33). See **Figure 1** for recruitment rates. The research team held a launch day at both study sites to inform service users and hospital staff about the IMPACT study and posters were displayed on the walls in the ward areas. Potential participants were screened by the clinical research officers from the Research Departments at both study sites after liaising with the responsible clinicians to ascertain whether participants were eligible to participate and if the research staff could approach participants to be involved in the study.

### Flow of participants from identification to entry into study

The numbers of people who were screened, eligible and consented to participate were recorded where possible. Eligible patients who did not wish to take part (i.e. unwilling to give consent) and those found to be ineligible went on to receive usual care from the service without prejudice.

### Ethics approval

This study was approved by the NHS North East – Newcastle & North Tyneside 2 Research Ethics Committee on the 21st of October 2021 (reference number 21/NE/0080) and the Health Research Authority (HRA) approval was obtained on the 30th of April 2021.

### Informed consent

Once eligibility was confirmed by the study setting, an IMPACT researcher arranged a convenient time to meet with the potential participant in the setting to discuss participation and provide information sheets. The first part of the meeting involved the researcher fully explaining the study and what would be involved (as per information sheet) and an opportunity for the person to ask questions and seek clarification. Written informed consent was then obtained, and baseline data was collected (or a further date was arranged for baseline data collection). The participants were given the intervention manual, which provided guidance and information about the IMPACT PA intervention. Participants were also given a copy of their completed consent forms.

### The IMPACT physical activity intervention

The overall aim of the intervention was for people in such settings to increase their physical activity (PA). The intervention was designed and co-produced with the service users and staff at both study sites, the research team and academics, using the theoretical framework of the Behaviour Change Wheel that is underpinned by the COM-B Model (4) and the Research Council’s framework for Developing and Evaluating Complex Interventions (18). The PA intervention was created to encourage service users to engage in PA in an independent and autonomous manner. The PA intervention considers the mental health, physical health and physical capability and accessibility of service users and supports service users to form achievable and personalised goals, which would result in encouragement and motivation to engage in PA. These elements were key discussion points when the PA intervention was designed and co-produced in Phases 1-3. The initial three phases of the IMPACT study are summarised below and in **Table 1**, a paper focusing on these Phases 1–3 is under preparation.

Phase 1: exploration of barriers and facilitators to increasing physical activity in medium secure psychiatric services.
Phase 2: investigation on how to establish engagement and maintain commitment to a PA intervention.
Phase 3: intervention development using the Intervention Mapping process (19, 20).

**Table 1:** Overview of phases 1–3 at both study sites.

Phase	Data collection method	Sample
**Phase 1** May 2022	Questionnaire with service users	**Site A:** 30 (25 male; 5 female) **Site B:** 30 (32 male; 6 female) Overall total *N* = 68
	Focus groups with staff	**Site A** *Focus Group 1* 2 Qualified Nurses. *Focus Group 2* 2 Ward Managers 2 Forensic Psychiatrists 2 Qualified Nurses. 1 Day Co-Ordinators **Site B** *Focus Group 1* 3 Day Co-Ordinators 2 Occupational Therapists 3 Sports and Exercise Facilitators 1 Research Fellow with expertise in physical and health *Focus Group 2* 2 Forensic Psychiatrists 1 Consultant Forensic Psychiatrist 1 Psychologist 2 Qualified Nurses. Overall total *N* = 24
**Phase 2** June 2022	Interview and focus groups with service users and key stakeholders	**Site A** *Focus Group 1* 2 Service Users *Focus Group 2* 2 Unqualified Nurses 3 Academics with expertise in physical health and/or behaviour change interventions **Site B** *Interview* 1 Service User *Focus Group* 2 Sports and Leisure Facilitators 1 Day Activity Co-Ordinator 1 Occupational Therapist 1 Research Fellow with expertise in physical and health 1 Consultant Forensic Psychiatrist 1 Trainee Doctor Overall total: *N* = 15
**Phase 3** Nov 2022	Intervention development groups	**Site A** *Group 1* 5 Service Users *Group 2* 2 Academics with expertise in physical health and/or behaviour change interventions 3 Unqualified Nurses 2 Student Nurses 1 Occupational Therapist 1 Sport Therapist **Site B** *Group 1* 5 Service Users *Group 2* 1 Consultant Forensic Psychiatrist 1 Psychologist 1 Sports and Exercise Facilitators 1 Research Fellow with expertise in physical and mental health 1 Unqualified Nurse 1 Primary Health Nurse Total *N* = 25

Phases 1–3 were conducted via focus groups and interviews with service users, hospital staff and academics that lasted up to 90 minutes and were held at both study sites. The research team used purposive sampling (21) to recruit participants to these focus groups, to allow for representation of different groups of service users and hospital staff. Four academics also participated with expertise in physical health, mental health and/or behaviour change interventions and were external to the research team. The co-produced PA intervention, from Phase 3, was designed by service users, hospital staff, research team members, Patient and Public Involvement and Engagement (PPIE representatives and academics after reviewing existing PA interventions and identifying elements that they felt would and would not work in a medium secure psychiatric setting. The Intervention Mapping Process (2, 19) was used to bring together existing evidence-based literature on PA interventions and the theory behind behaviour change, to support the development of this co-developed PA intervention. Once individual aspects of other existing, successful PA interventions were identified, it was then mapped to the COM-B model (22) to form the basis of the PA intervention. Aspects of the PA intervention addressed the three factors of the COM-B model (Capability, Opportunity and Motivation). For example, discussions were held around the lack of staffing and staff capacity to support with PA interventions, which affects service users’ physical opportunity to engage in PA. Thus, the IMPACT PA intervention was developed to utilise the existing resources provided by the hospital and for service users to take on an autonomous and independent approach when engaging and tracking their PA levels. Staff were still involved and encouraged service users who participated in the PA intervention but a key aspect that was discussed when defining the role of hospital staff in this PA intervention, was that the intervention should try and prevent from adding onto day-to-day clinical procedures and hospital staffs’ existing heavy workload.

Another aspect that was discussed and mapped onto the COM-B model was that service users and staff were concerned about participants cognitive capability in engaging in a PA intervention. For example, symptoms of mental illness or side effects of medication. The IMPACT PA intervention took this into consideration and promoted an individualised and graded approach to engaging in PA. Participants who felt more capable were able to choose a group that encouraged more minutes of PA per week, whereas participants who felt that they needed to start slowly chose the ‘starting to be active’ group. At the end of the intervention development group in Phase 3, the IMPACT PA intervention manual was developed, which provided guidance and information for participants and hospital staff.

### Intervention content

The PA intervention was designed to be delivered for 3 months (12 weeks) during Phase 4. At commencement, the research team introduced the IMPACT Intervention to hospital staff and service users with an awareness discussion about PA and health lifestyles. In the introductory sessions, the points below were discussed:

1. The barriers and facilitators of increasing PA in medium secure services.
2. The level of PA that participants/service users should be engaging in, in accordance with NHS guidelines.
3. The PA Intervention and how it will operate.
4. Staff support and encouragement throughout the intervention.

The participants who consented to participating had an initial meeting/assessment with the research team to ensure that they understood the intervention, what personal goals they wished to achieve, a discussion about their current level of engagement in PA and whether they were interested in access to a pedometer. The responsible clinician to each participant was consulted about their eligibility in accessing a pedometer based on individual risk assessments. The hospital staff and participants were given an intervention manual, which explained the PA intervention and included materials to support the intervention.

In the initial meeting/assessment, the participant decided on which one of three PA groups, in accordance with NHS recommendations (23), they wanted to be categorised into:

1. Starting to be Active - aim to engage in 60 minutes of PA a week.
2. Intermediate - aim to engage in 150 minutes of PA a week.
3. Pushing the Limit - aim to engage in 180+ minutes of PA a week.

The research team encouraged the participants to engage in a variety and novel types of PA, for example, gardening, cleaning, walking around the ward area, and not to feel that the definition of PA was limited to using the gym. The research team asked hospital staff to offer support, encouragement and prompts for participants. Once the participant started the PA intervention, the research team organised regular weekly visits with the participant to speak to them about the PA activity levels. Participants were asked to keep a weekly record of their engagement in PA, which included what type of PA they engaged with, whether this was on their own or with peers, and how long they engaged in PA.

### Outcomes

The main outcome of the IMPACT study was to establish the feasibility and acceptability of an evidence-based, co-produced intervention to promote physical activity. Qualitative data on acceptability, and quantitative data on recruitment, participation, retention and causes of drop-out were collected. This paper focuses on a descriptive analysis of the quantitative data outcomes. Another paper focusing on the qualitative feedback from service users and hospital staff regarding the IMPACT PA intervention is under preparation.

All outcome measures were collected at baseline (prior to the intervention), post-intervention (after the intervention) and at follow-up stage (three months post intervention).

#### Primary outcome measures

One of the primary outcome measures was the International Physical Activity Questionnaire - Short Form (IPAQ-SF) (24) which has been validated for use among individuals with severe mental illness (25, 26). The IPAQ asks for information on participants’ PA levels in terms of intensity. There are 7 items that require participants to state the number of days per week and time they spent engaging in vigorous PA, moderate PA, walking and sitting. The results from the IPAQ-SF were analysed based on the reported time spent engaging in moderate physical activity (MPA) and vigorous physical activity (VPA). The calculations for MPA used the time spent engaging in MPA multiplied by the number of days spent engaging in MPA (25). The calculations for VPA used the time spent engaging in VPA multiplied by the number of days spent engaging in VPA. MVPA (Moderate to Vigorous Physical Activity) was calculated by adding the MPA and VPA scores.

Another primary outcome measure was the Single Item Self-Assessment. The participants were asked to self-assess whether they felt that they had achieve the NHS target/recommend levels of 150 minutes of PA per week, enough to raise their breathing rate and includes sport, exercise, brisk walk or cycling. This single-item question was first devised and validated by Milton et al. (27) and a review showed that that it is a useful tool to detect change in PA levels (28). Participants were asked in the past week, on how many days have you done a total of 30 mins or more of physical activity, which was enough to raise your breathing rate? This may include sport, exercise and brisk walking or cycling for recreation or to get to and from places.

#### Secondary outcome measures

- Behavioural Regulation in Exercise Questionnaire (BREQ-3) (29): This is a 24-item questionnaire that measures different aspects of participants’ motivation and regulation in behaviour related to exercise and physical activity. These include Amotivation, External Regulation, Introjected Regulation, Identified Regulation, Integrated Regulation and Intrinsic Regulation and scoring identifies participants’ degree of self-determination. Participants are asked to read statements such as ‘I exercise because it is fun’ and rate on a scale (0 = not true for me, 2 = sometimes true for me, 4 = very true for me).
  To investigate participants’ degree of self-determination, the relative autonomy index for BREQ-3 was used. The relative autonomy index provides one score that indicates the level of relative autonomy and self-determination. To calculate this score, once the individual subscales have been calculated, it is then multiplied by a weighting (Amotivation *-3, External Regulation*-2, Introjected Regulation*-1, Identified Regulation*1, Integrated Regulation*2 and Intrinsic Regulation*3), and then summed for the final relative autonomy index.
- Warwick–Edinburgh Mental Wellbeing Scale (WEMWBS) (30): This is a self-reported questionnaire to measure well-being. There are 14 items and participants are asked to read statements such as ‘I’ve been feeling relaxed’. Participants then rate the statements on a scale (1 = none of the time, 2 = rarely, 3 = some of the time, 4 = often, 5 = all the time). Blood pressure was measured regularly at both sites as a standard of practice. The research team were able to collect this data from the hospital information system.
- Body fat (%) and muscle mass (kg) were measured using the Tanita Machine, a body composition scale (31).
- The Home Step Test (HST) (32) asked participants to step up and down a step for 3 minutes at a consistent pace. Their heart beats per minute were then collected after this exercise by the research team.

### Acceptability

In both study sites, all intervention service users and 6–10 staff members with direct participant contact were invited to a 1:1 semi-structured interview (up to 30 minutes) Service user interviews explored intervention acceptability and perceived effectiveness (e.g., usefulness, impact on daily life, feelings, and mood). Staff interviews focused on the intervention’s acceptability and its perceived effects on participants’ well-being, quality of life, and everyday functioning. All interviews took place after intervention completion.

### Intervention completion and exit

The research team considered participants as having exited the intervention when they:

- completed the intervention;
- expressed that they wished to withdraw consent; or
- had their consent withdrawn by the research team or clinical team due to risk of harm to self or others.

### Withdrawals

Participants could withdraw from the study at any time and were aware that the data up until the point of them withdrawing would be retained unless otherwise requested. The research team could also withdraw participants from the study if it negatively impacted the participants’ mental health or treatment plan, or if risk/harm to themselves or others was identified. The participants received a thank you letter for their participation from the research team. See **Figure 1** for more information on the withdrawal rates of participants.

### Adverse events

Adverse events were monitored by the Research Management Group (RMG) and the Research Steering Group (RSG). In the occurrence of a serious adverse event (SAE) related to the study, the RMG and RSG would immediately be notified and asked to review any reported SAEs that were deemed to be study and/or intervention related. No adverse events or incidents occurred to participation in the study.

### Data analysis

As this was a feasibility study, no inferential analysis was undertaken to test for intervention effectiveness therefore all statistical analysis was descriptive. The primary focus is on summaries of key indicators of success of the study and data is reported according to the CONSORT extension to Randomised Control Trials (26) including total numbers recruited and consented; participants completed baseline questionnaires and who received the intended intervention; retention and who were assessed at follow-up (including any reasons for loss to follow-up). The flow of participants is detailed in **Figure 1** and **Table 2**.

**Table 2:** Participant Demographics across both study sites.

Characteristic	Site A	Site B	Total
Age			
Mean (SD)	39.56 (6.73)	39.06 (12.07)	39.23 (10.38)
Range	30 to 52	21 to 65	21 to 65
Gender			
Male	5 (56%)	14 (82%)	19 (73%)
Female	4 (44%)	3 (18%)	7 (27%)
Ethnicity			
Asian or Asian British	0	1 (5.9%)	1 (3.8%)
Black, African, Caribbean, or Black British	0	1 (5.9%)	1 (3.8%)
Mixed or Multiple Ethnic Groups (White and Black Caribbean/White and Black African/White and Asian	1 (11.1%)	3 (17.6%)	4 (15.4%)
Any other Mixed or Multiple Ethnic Background	1 (11.1%)	0	1 (3.8%)
White	6 (66.7%)	9 (52.9%)	15 (57.7%)
Other Ethnic Group	1 (11.1%)	1 (5.9%)	2 (7.7%)
Do not know or prefer not to say	0	2 (11.8%)	2 (7.7%)
Length of Admission			
Total			
Mean (SD)	1.83 (1.78)	3.79 (3.91)	3.09 (3.40)
N	9	16	25
Female			
Mean (SD)	2.00 (1.80)	3.28 (4.11)	2.55 (2.78)
N	4	3	7
Male			
Mean (SD)	1.69 (1.97)	3.91 (4.03)	3.29 (3.66)
N	5	13	18
Smoking History			
Total (N)			
Yes	6 (35.29%)	11 (64.71%)	17 (65.38%)
No	3 (33.33%)	6 (66.67%)	9 (34.62%)
Female (N)			
Yes	3 (50%)	3 (50%)	6 (23.08%)
No	1 (100%)	0 (0%)	1 (3.85%)
Male (N)			
Yes	3 (27.27%)	8 (72.73%)	11 (42.31%)
No	4 (57.14%)	3 (42.86%)	7 (26.92%)

GRL transcribed the qualitative data verbatim from audio recordings and transcriptions were read multiple times by GRL to allow familiarity of the data. Framework analysis (18) was used by GRL to analyse the interviews.

## Results

### Recruitment

The original recruitment target for testing feasibility and acceptability of the PA intervention was 15–20 participants at both study sites (n=30-40), over 3 months of recruitment. However, prior to recruitment, the research team had an in-depth discussion with each study site to understand their capacity and how many participants they could recruit. Study Site A stated that due to lack of staff support, they could potentially recruit up to 10–15 participants and Study Site B suggested recruiting up to 20 participants. In total, the research team recruited 10 participants at Study Site A and 23 participants at Study Site B (n=33). Recruitment was completed in-person with service users on the ward they resided on.

### Follow-up, withdrawals, and intervention attendance

There was good retention in the study. Overall, twenty-six participants completed the PA intervention and only 7 participants withdrew during the intervention period (n=1 at Study Site A; n =6 at Study Site B). Three participants withdrew at the start of the intervention (N =1 at Study Site A and N =2 at Study Site B), and this was due to a deterioration in their mental health and wellbeing. They withdrew after the baseline measures were completed and did not start the PA intervention. One participant withdrew towards the end of the PA intervention due to a deterioration of their mental health and wellbeing. Two participants withdrew in the middle of the PA intervention due to being transferred prison from hospital/transferred to a different hospital. One participant withdrew towards the end of the PA intervention due to their physical health.

During the follow up stage, three months after the intervention, 3 participants withdrew (n=2 at Study Site A; n=1 at Study Site B). Reasons for withdrawing from the follow up stage were due to mental health deterioration. However, overall, there was a good retention of participants at the follow up stage, three months after the intervention was completed (n=22; n = 7 at Study Site A and n = 16 at Study Site B). **Table 2** presents the demographics of the participants in the study.

### Primary outcome measure

The results for MVPA from the IPAQ-SF (24) for all three time points, baseline, post intervention and follow up are displayed in **Table 3**.

**Table 3:** Primary and secondary outcome measures across three time points.

Outcome measures	Timepoints		
	Baseline	Post intervention	3-months post intervention
IPAQ-SF: Moderate and vigorous physical activity (MVPA)			
Mean (SD)	135.60 (135.26)	185.96 (184.28)	253.13 (180.43)
N	25	26	8 (23)
Did Participants achieve NHS target of 150 mins of physical activity/week?			
Yes	4	12	8
No	18	12	15
BREQ-3 (relative autonomy index)			
Mean (SD)	50.27 (23.31)	52.54 (18.80)	52.09 (20.67)
N	26	26	23
WEMWBS			
Mean (SD)	50.00 (9.45)	51.77 (9.69)	47.87 (10.04)
N	25	26	23
Blood pressure			
Systolic			
Mean (SD)	133.50 (21.14)	122.92 (14.83)	128.43 (15.28)
N	16	24	23
Diastolic			
Mean (SD)	85.31 (12.02)	81.75 (9.77)	84.70 (9.95)
N	16	24	23
Body fat (%)			
Mean (SD)	38.66 (14.13)	38.20 (12.29)	37.09 (12.70)
N	23	22	19
Muscle mass (Kg)			
Mean (SD)	56.93 (4.54)	58.11 (3.30)	57.98 (6.92)
N	23	22	19
Home step test (bpm)			
Mean (SD)	110.00 (27.31)	102.00 (23.66)	104.76 (16.37)
N	21	4	17

The number of participants in IMPACT was small, however, it can be seen from **Table 3** that there does appear to be an increase in self-reported MVPA from baseline (prior to the intervention) and post intervention (after the intervention) in our population for those who received the intervention.

At the follow up stage (three months post intervention) of the study, all 23 participants completed the IPAQ-SF. However, only eight out of the 23 participants provided an answer to the MVPA questions. This was potentially due to participants not completing the full questionnaire and the scoring method of the IPAQ-SF. If a participant omits a question, this will result in that answer being coded as ‘missing’, even if the participant were to answer other questions on the IPAQ-SF. Thus, participants who completed the IPAQ-SF at the follow up stage (three months post intervention) but did not offer information about their MVPA are not included in the total n value. This is an important factor to note as the research team found that the participants were keen to participate in the follow up stage of the study, but the IPAQ-SF scoring method would indicate otherwise based on face value. However, based on **Table 3** and the participants who did answer the IPAQ-SF, participants self-reported more MVPA compared to baseline. See **Table 3** for more information across all time points.

The participants were asked to self-assess whether they perceived that they had achieved the NHS target/recommend levels of 150 minutes of PA per week (23), enough to raise their breathing rate and includes sport, exercise, brisk walk or cycling. The results suggest that from baseline (prior to intervention) to post intervention (after intervention), showed more participants self-reported that they were achieving the target of 150 minutes of PA per week. At baseline, only four participants reported they were achieving the 150 minutes of PA per week target but at the post intervention stage, 12 participants felt that they were achieving this. See **Table 3** for more information across all time points.

### Secondary outcome measures

The BREQ-3 (29) asked the participants to self-report their motivation and amotivation about exercise. Results at baseline (prior to intervention, m =50.27) to post intervention (after intervention m = 52.54), suggest that there is a trend towards a higher degree of self-determination that is maintained at follow up (three months post intervention, m = 52.09). See **Table 3** for more information across all time points.

The WEMWBS (30) measured the mental wellbeing of the participants and results from baseline (prior to intervention, m =50.00) to post intervention (after intervention m = 51.77), suggest there was a positive increase in mental wellbeing. However, the WEMWBS scores decreased at the follow up stage (three months post intervention). See **Table 3** for more information across all time points.

### Blood pressure

The results suggest a decrease for both systolic and diastolic blood pressure from baseline (prior to intervention) to post intervention (after intervention). Across both study sites, at baseline (n = 16) the systolic blood pressure averaged at M= 133.50, SD = 21.14 and diastolic blood pressure averaged at M = 86.31, SD = 12.06. At post intervention (n=24), systolic blood pressure average at M = 122.91, SD = 14.83 and diastolic blood pressure averaged at M = 81.75, SD = 9.77. However, both systolic and diastolic blood pressure increased at the follow up stage (three months post intervention). The systolic blood pressure at the follow up stage (n = 23) averaged at M= 128.43.50, SD = 15.28 and diastolic blood pressure averaged at M = 84.70, SD = 9.95. See **Table 2** for more information across all time points.

### Body fat (%)

The results do not suggest change for body fat from baseline (n=23, m =38.66%), the intervention period (n = 22, m = 38.20%) or the follow up stage (n=19, m = 37.09%). See **Table 3** for more information across all time points.

### Muscle mass (kg)

The results suggest an increase in muscle mass (kg) for the participants (n=23) from baseline (prior to the intervention, m = 56.93kg) and post intervention (n22; m = 58.11kg). See **Table 2** for more information across all time points.

### Home step test

The HST results suggest a decrease in heart beats per minute over the three time points. However, it is important to note that there were difficulties in the recruitment of participants to complete the Home Step Test. Therefore, the results for the HST must be interpreted with caution. See **Table 3** for more information across all time points.

### Acceptability

Twenty-six service users (Study Site A n=10; Study Site B n= 16) and 13 staff (Study Site A n=7; Study Site B n= 6) completed the acceptability interviews. Qualitative results suggested enthusiasm and satisfaction for the intervention, a desire to continue exercising after the study and positive feedback and suggestions were offered to help inform future work in this area. See **Table 4** for some preliminary quotes on acceptability. A qualitative paper is currently being produced.

**Table 4:** Quotes from acceptability interviews phase 4.

Participants	Quotes
Service Users	*N001: Really enjoyed intervention, made me feel motivated and gave me a boost.* *A074: Found myself pushing to do that extra minute of walking or extra bench at the gym.* *N010: My mood has improved, and it was nice to set a target and keep at it.*
Staff	*A002: Service users commented that they have more energy and felt less depressed.* *A003: Intervention was very useful as it motivated and engaged patients who otherwise wouldn’t do anything.* *N002: Allowed service users to gradually increase their exercise.*

## Discussion

The IMPACT study was the first UK-based investigation to test the feasibility of co-producing an evidence-based intervention to increase PA in medium secure settings. To our knowledge no other studies related to this area have been conducted in the UK. As well as identifying that such a study is acceptable and feasible, it also has provided useful insights on recruitment, data collection, and outcomes measures for a more robust clinical trial in the future. The challenges and solutions that have been identified are likely to be applicable to further sites in a larger pilot RCT trial.

The study aimed to establish feasibility and was not powered to detect statistically differences, as such, no inferential analysis was undertaken. The research team were able to collect in-depth quantitative data and gain contextual information about both study sites. The target of 40 was not achieved due to staffing capacity and workload at Study Site A. However, thirty-three people with SMI across the two medium secure study sites in England did engage with the study and this is a positive finding, especially as we identified factors affecting recruitment and retention. Retention was good and 26 participants completed the PA intervention and only 7 participants withdrew during the intervention period.

At the end of the intervention period, self-reported physical activity and physical health outcomes were higher following the IMPACT intervention. A significant impact from the results is that the PA intervention was associated with an increase in the primary outcome - increased self-reported physical activity. The IPAQ-SF suggested an increase in MVPA levels, and the self-assessment single item question suggested that more participants perceived that they were achieving the recommended 150 minutes of PA per week. This is a positive signal as to the potential of the intervention. However, due to the smaller sample size, a more rigorous research design using additional measures of physical activity, whether device-based or through direct observation, will be needed to confirm this potential. Although, in the IMPACT PA intervention, initially pedometers were offered to participants, due to restrictions and other personal reasons from the participants, the uptake of pedometers was not high. Perhaps it would be important to determine what type of device is best suited for secure psychiatric settings.

An outcome measure with questionable acceptability and suitability for the PA intervention in this setting was the HST. Many participants declined to engage with this outcome measure, citing physical health problems and disinterest in completing it. In addition, with the complex nature of the environment, it was often difficult to access steps to administer the HST. For example, at Study Site B, during the post intervention stage, there were difficulties with staffing at the hospital to support with the HST. Therefore, the HST was only completed at two time points at Study Site B and three time points at Study Site A. Thus, the results of the HST should be interpreted with extra caution due to the low numbers of participants completing it. Furthermore, this does show that perhaps there are practical issues which make it difficult to administer the HST in secure psychiatric services and it may be necessary to explore other measures to investigate cardiovascular health in this setting. However, the HST was initially planned to measure the effectiveness of the PA intervention, as increased PA over time should enable an increased performance whist completing the HST. Therefore, it may be that a variety of other measures of PA performance need to be explored and considered to better suit the participants in this setting. For example, the 6-minute walk test or the self-paced walk test etc (33, 34),. The results showed a reduction in the blood pressure in participants at post intervention, compared to baseline levels. However, blood pressure can be seen to have fluctuated and increased at the follow up stage. It could be argued that blood pressure tends to fluctuate depending on the time of day it was measured and the medication that the participants were taking for their SMI. The continued use of the Tanita machine that measured body fat (%) and muscle mass (kg) will need to be carefully considered in a future study. The research team noted that there was a negative connotation used by the participants regarding the machine and it was labelled as the “fat machine.” Further, there were concerns raised by the clinical team at the study sites regarding existing body image problems that participants may have and the use of the machine.

In the results, there was an indication of a positive impact on participants’ mental health, according to the WEMWBS, which corresponds with the literature that identified PA as a mental health promotion strategy (35). The research team noted a change in attitude and views at the hospital, where more people were talking and engaging in physical activity, and this could be a focus for evaluation in a future study. This will be discussed in more detail in a qualitative paper on the feedback from service users and hospital staff which is currently in preparation.

However, although initially there was a positive impact on mental health, the results suggest that at the follow up stage (three months post intervention), there was a decrease in mental wellbeing, lower than the levels of baseline (prior to the intervention). The research team were able to collect contextual information on changes in the ward environment that may be insightful to explain this decrease in mental wellbeing, such as deterioration of mental health, serious incidents of self-harm and staff shortages, which were all unrelated to the study. In addition, the mean score for the WEMWBS for the UK general population is 51.0, which is unlikely to be clinically different from the follow up stage (three months post intervention) score of 47.87, factoring the population and setting.

Nevertheless, another potential explanation for the reduction in wellbeing scores is that the ‘withdrawal of exercise’ can increase symptoms of mental health issues. In a systematic review in 2017 it was concluded that withdrawal of exercise can increase the symptoms of depression and anxiety (9). This point further highlights the importance of PA and its associations to mental health wellbeing, which aligns with the results that show an increase in mental wellbeing at post intervention.

Weinstein et al. (9) further noted that ‘withdrawal of exercise’ needs to be taken into consideration for future research and clinical interventions. In the IMPACT study, post intervention and at the follow up stage, the research team reminded participants to continue tracking their PA levels, using the materials given to them during the intervention such as the weekly tracker. Ideally for future interventions, a statement for the participants on what to expect after the intervention is complete, may help mitigate the negative impact that comes from ‘withdrawing’ from the participants or the intervention period coming to an end. Our positive acceptability results provide preliminary evidence that there was enthusiasm and satisfaction for the intervention, and it improved exercise and mood in service users, however this was not assessed objectively.

In conclusion, the study was able to recruit a sample of people who are living with SMI in medium secure psychiatric services and retain them in both the intervention and data collection. There was a signal that the intervention might have a positive effect as indicated by a self-reported increase in PA after the intervention. Patients and staff reported the intervention as acceptable. Based on these findings we have concluded the intervention is feasible and acceptable. This study will form the basis for planning a future pilot RCT with a view to scaling ultimately across other medium secure psychiatric services in the UK.

## Data Availability

The original contributions presented in the study are included in the article/supplementary material. Further inquiries can be directed to the corresponding author.
